# Driving Mechanism for Manufacturer’s Decision of Green Innovation: From the Perspectives of Manager Cognition and Behavior Selection

**DOI:** 10.3389/fpsyg.2022.851180

**Published:** 2022-03-28

**Authors:** Minghua Han, Daliang Zheng, Danyi Gu

**Affiliations:** Business School, Ningbo University, Ningbo, China

**Keywords:** manufacturer, green innovation, decision, mechanism, embodied cognition, behavior selection

## Abstract

From the perspectives of manager cognition and behavior selection, this paper analyzes the cognitive basis of manufacturer’s green innovation and discovers that the embodied cognition of the manager has an important influence on the selection of green innovation behavior. Next, the behavior activation in the four stages of manufacturer’s green innovation, namely, initiation, termination, change, and solidification, was analyzed, and two behavior selections were proposed: the adaptive legitimacy with institutional logic as the cognitive starting point and the strategic legitimacy with efficiency logic as the cognitive starting point. On this basis, the authors examined four types of manufacturer decisions of green innovation (compliance, selection, creation, and control) driven by manager cognition and behavior selection. The examination reveals how should the manager, facing the growing environmental pressure, form a correct cognition, select the right behavior, and make the proper green innovation decision, which promotes the green, sustainable development of manufacturers.

## Introduction

Green innovation achieves sustained economic and environmental performance by reducing the full-lifecycle eco-environmental effects of products (Chen, 1999; Xiang et al., 2002). It is an innovation that significantly benefits the environment (Driessen and Hillebrand, 2002). Despite enabling enterprises to form dynamic green capacity (Huang et al., 2015), green innovation faces problems, such as heavy investment cost, long return period, and high risks (Li, 2019). As a result, manufacturers are often caught in a dilemma, when they make the decision on whether to adopt green innovation.

On the driving factors of corporate green innovation, most scholars held that external forces play an important role in the corporate decision of green innovation, such as market drivers and government environmental regulations. In the real world, green innovation drivers also exist within the enterprises (Xie et al., 2019). These internal factors fall on the organizational level and individual level (Peng and Huang, 2013; Zhao and Zhang, 2019; Wei, 2020). The existing studies have provided a series of meaningful results on how manufacturers make green innovation decisions under the joint effect of internal and external drivers. However, there are two main defects: (1) some studies fail to classify the target industries and (2) the studies rarely consider the mechanism of individual cognition and behavior logic of the manager acting on the decision of a manufacturer about green innovation. In fact, the decision of a manufacturer about green innovation is closely associated with the cognitive level and behavior selection model of corporate managers (senior executives). Therefore, this paper explores the driving mechanism for manufacturer’s decision of green innovation, from the angles of manager cognition and behavior selection. The research results provide a reference for manufacturers to realize high-quality development through green innovation.

## Cognitive Basis of Manufacturer’s Green Innovation

### Manager Cognition

The manager cognition refers to the knowledge combination underpinning the decision of the corporate managers (Borkent, 2015), which supports the decision of a manager through information identification, interpretation, and action. In cognitive psychology, individual’s cognition is usually explained in the following aspects by the concept of embodiment (Borkent, 2015), which in the body is an important factor that affects individual’s cognition, and the body acts as the carrier of individual behavior. Each individual has a unique perception and experience of the surrounding environment. Thus, the embodiment of physical experience leads to the difference in individual’s cognition (Ye, 2014). The various events and physical processes in the external environment are the cognitive resources of subjective initiative (Spackman and Yanchar, 2014). In the presence of these cognitive resources, individuals are willing to find solutions based on the existing resources in a particular situation. Whether to possess resources, whether they are willing to use resources, and how to utilize resources depend on the previous experience of the individuals.

In fact, the individual’s cognition formed in a certain environment tends to stagnate, unless disruptive changes take place in the environment (North, 1990). As a result, corporate managers easily ignore changes in the environment, making it difficult for enterprises to make suitable decisions in the face of various dynamic environmental changes.

### Manufacturer’s Green Innovation

Green innovation has a positive impact on the sustainability of manufacturers. In general, manufacturer’s green innovation can be divided into green process innovation and green product innovation (Xie and Zhu, 2021). The former is mainly the innovation of the production end. The latter targets the entire production cycle. The focus of manufacturer’s green innovation varies with the innovation orientations. To reduce the environmental pollution and resource waste of the production process, green process innovation of manufacturers stresses the reform of the local production model of enterprises. In essence, green process innovation attempts to eliminate or reduce pollution throughout the production process and ensure that corporate development is in line with environmental policies. Green product innovation of manufacturers emphasizes the reform of the value chain. In essence, green product innovation aims to produce greener and more environment friendly products and to bring differentiated advantages for enterprise products.

### Manufacturer’s Green Innovation Under Manager Cognition

In the market economy, an enterprise has two attributes, namely, economic man and moral man (Zhu, 2014). An externality exists through the production and operation of enterprises. Hence, there might be contradictions and conflicts of interest between the profit-seeking nature of enterprises and the social benefits (e.g., eco-environmental protection) (Xu, 2021). It is a major challenge for enterprises to realize harmonious coexistence with the environment. For manufacturers, the handling of the challenge is closely associated with the cognition of the manager (especially senior executive): whether green innovation is considered in decision-making and whether the enterprise would carry out green innovation activities. The reason is that the previous experience of production and operation shapes the personal cognition of corporate managers (especially senior executives), which determines their thinking pattern, and ability to accept and judge information. The personal cognition helps corporate managers (especially senior executive) to recognize and determine the external environment of the enterprise and promotes them to change or adjust the production and operation field or direction of the enterprise, according to the variation in the external environment.

During the change or adjustment, the cognition of the corporate managers (senior executives) also changes. However, the change is bounded by the limitation of the embodied cognition of corporate managers (senior executives) (Ye, 2014). That is, the decision path and behavior pattern are still based on the previous cognitive architecture, without breaking the original thinking pattern or cognitive system. In the face of green innovation, which demands conceptual reform, the manufacturer must fundamentally change its original operation philosophy. Meanwhile, the embodied manager (senior executive) of the manufacturer, which is seamlessly integrated into the environment, needs to break away from the innate cognition and realize the importance of reducing negative environmental impacts through green innovation under the interaction between internal psychology and external environment (Zhang and Li, 2021). In this way, the manager (senior executive) will be more willing to pursue green innovation and promote the manufacturer make behavior selection to put green innovation into practice. Therefore, the manufacturer’s behavior selection of green innovation depends heavily on the environmental cognition of the manager (senior executive).

## Behavior Selection Analysis of Manufacturer Green Innovation

### Activation Process of Manufacturer’s Green Innovation Behavior

The analysis in section Manufacturer’s Green Innovation Under Manager Cognition shows that manufacturer’s green innovation requires the corporate managers (senior executives) to transform the original cognition, establish the corresponding cognition of the environment, and further convert the cognition into green innovation practice. That is, the manager must complete the shift from the self-enhancement model to problem-solving model. The psychological features of the latter model promote the managers to reform their cognition (Shang et al., 2014). The shift covers four stages.

(1) Initiation stage: The changes in external environment activate the cognitive reform of the management. The cognition reform of the manager starts from the changes in the external environment of the enterprise. The environmental factors exert constitutive effects on the embodied cognition of the manager (Ye, 2014). Based on the perception of the external environment and the *status quo* of corporate development and operation, the manager evaluates the living environment and potential opportunities or risks facing the enterprise. If the other similar enterprises in the same industry respond and adjust timely (e.g., adopting green innovation) to cope with environmental pressure, the manager will realize that the living environment of the enterprise has been fundamentally changed. Bearing this in mind, the managers will regulate their mental state and change their cognition, break away from the original thinking pattern, and try to find the right solutions from different angles and directions. Meanwhile, the manager will incorporate the following into individual cognitive systems: the concerns of environmental changes and the understanding of problems of external stakeholders, who are affected by organizational decision and behavior. Hence, the changes of the external environment, which is critical to the survival and development of the enterprise, could stimulate the managers to complete the psychological shift toward the problem-solving model and reform their cognition of green innovation.
(2) Termination stage: The management denies its own cognition and triggers trial-and-error learning. Once the cognition reform of green innovation is activated, the behavior of the manager will change: an exploration will begin concerning the direction of corporate green innovation. However, this does not exceed the original scope of manager cognition. Only when problems are detected through the exploration, the managers will doubt and deny their perception of green innovation. It signifies the termination of the originally embodied cognition. In other words, when profound changes of the external environment bring severe challenges and major problems to the enterprise, the manager tends to look for pertinent solutions, e.g., green innovation practice, to problems of corporate development, based on the scope of the original cognition. If negative feedbacks occur, the managers will attribute the failure to the bias and even incorrectness of the original cognitive scope, deny embodied cognition, and become willing to reform their cognition of green innovation.
(3) Change stage: The management constructs green innovation cognition through trial-and-error learning. When the manager tries to solve the corporate development problems brought by external environmental pressure through green innovation, the managers would continuously examine the specific causes of new problems arising during the solution of the current problem and adjust their cognition accordingly. Meanwhile, new attempts are made to solve the new problems. The trial-and-error and learning process are implemented iteratively until all problems are truly solved. Each round of trial-and-error and learning reshapes the original cognition of the manager. Through continuous adjustment, the embodied cognition of the manager rises in a spiral. In this process, the originally embodied cognition of the manager is gradually phased out, and a new cognition is constructed to ease external environmental pressure and implement green innovation. The new cognition paves the way to green innovation decision by the manufacturer.
(4) Solidification stage: The management forms a new cognition theory and externalization. Through a loop of trial-and-error learning of the third stage, the new embodied cognition formed in the change stage is natural legitimacy and compliance. Therefore, the manager will solidify the new embodied cognition through theorization. At the same time, after the completion of the reform of embodied cognition, and the solidification of green innovation cognition, new changes take place in the management behaviors around corporate green innovation. It is necessary for the manager to implement the green innovation cognition in practice. Putting the cognition into practice promotes manufacturer’s green innovation, turning green innovation into a habit of the enterprise.

### Behavior Selection of Manufacturer’s Green Innovation

Based on the organizational level, the green innovation behavior of manufacturers manifests the corporate compliance with the concept of green development. The behaviors are in line with the values, goals, norms, concepts, and needs of ecology first and green development and conducive to the harmonious coexistence between the enterprise and the eco-environment. In this way, the enterprise can solicit the support and recognition of the government, the market, and consumers, by virtue of its legitimacy. This proactive green innovation behavior could be affected by the embodied cognition of the manager (especially senior executive). Under different embodied cognitions, the manufacturer selects different green innovation behaviors to win legitimacy. There is the adaptive legitimacy with institutional logic as the cognitive starting point (Xie and Zhu, 2021), and the strategic legitimacy with efficiency logic as the cognitive starting point (Xie and Zhu, 2021).

The adaptive legitimate behavior selection for green innovation emphasizes that manufacturers implement green innovation out of compliance and believes that the survival and development of manufacturers depend on the market and their institutional environment (Scott, 1995). Complying with the institutional environment is the only source of legitimacy of the enterprise. On this basis, manufacturers will mainly follow the institutional logic (Xie and Zhu, 2021) in green innovation behavior. To pursue legitimacy, the enterprise would adopt a passive strategy dominated by the compliance with institutional control. The strategy highlights the compliance with industry regulations and codes of conduct. In the end, all enterprises in the industry will adopt the same logical strategy, i.e., their behaviors will converge. The positive signals conveyed during the period enable enterprises to gain recognition from external stakeholders and provide them with the resources needed for innovation. Then, the enterprises can transform resources, information, knowledge, institutions, and norms into green innovation capacity and pursue green innovation under the incentive of institutional logic, thereby alleviating the pressure of the external environment.

The strategic legitimate behavior selection for green innovation emphasizes that enterprises carry out green innovation by their subjective initiative and believes that the continuous growth of the manufacturer depends on its resource integration capacity. The enterprise should effectively integrate its own resources with externally acquired resources, overcome its resource limitation, and seek new opportunities to promote rapid development. On this basis, manufacturers will mainly follow the efficiency logic (Xie and Zhu, 2021) in green innovation behavior. That is, the enterprise will use legitimacy as an important means of obtaining resources. Besides obeying environmental laws and regulations, the enterprise will actively exert its own initiative and take a proactive approach to obtain resources as soon as possible. Then, the resources will be integrated and optimized to maximize the resource advantage. In this way, the enterprise can lead competitors by a wide margin and receive the environmental premium. In this process, the manufacturer actively performs related environmental and social responsibilities and provides green products, which are expensive yet attractive to consumers, aiming to gain a sustainable competitive advantage.

Hence, legitimacy provides the criterion of corporate managers (especially senior executives) to judge and choose embodied cognition and determines the behavior selection for manufacturer green innovation.

## Manufacturer’s Decision of Green Innovation Driven by Cognition-Behavior Selection

**FIGURE 1:**
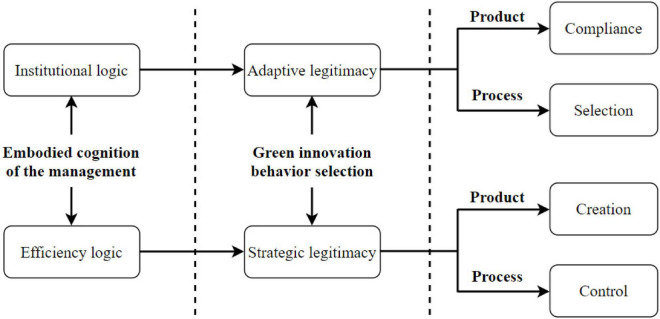
Manufacturer decision of green innovation driven by cognition-behavior selection.

The previous analysis shows that, firstly, managers (especially senior executives) form corresponding embodied cognition under the pressure of the external environment. Secondly, the cognition is transformed into green innovation behavior selection of a different manufacturer (adaptive legitimacy and strategic legitimacy). On this basis, finally the difference in behavior selection results in varied green innovation decisions. Even if the behavior selection is the same, the green innovation decision may vary, owing to the disparity in the problem-solving method and process (green product innovation or green process innovation). Figure 1 shows the manufacturer’s decision of green innovation driven by cognition-behavior selection.

As shown in Figure 1, the managers (especially senior executives), after selecting adaptive legitimate behavior of green innovation under the cognition of institutional logic, will make one of the two different green innovation decisions, namely, compliance and selection, in view of their cognition of institutional pressure, during the acquisition of relevant resources and maintaining legitimacy through green innovation. Specifically, compliance is the green innovation decision of the manager, upon perceiving strong pressure from external environmental changes. The manager decides to obey government systems and industry practices. Under the premise of respecting nature and making full use of natural resources, the manufacturer will work to reduce the environmental pollution that may occur during the product lifecycle, and new green products of a manufacture that meet environmental requirements and are harmless or minimally harmful to the environment. Selection is the green innovation decision of the manager, after fully considering the institutional pressure in the environment and the situation of corporate development. Without sacrificing the production capacity, the enterprise chooses to optimize and adjust some links in the production process, trying to reduce the production pollution and the generation of hazardous waste, lower the pollution discharge to the level below relevant laws, regulations, and standards, and achieve clean and up-to-standard production. Under the cognition of institutional logic and the selection of adaptive legitimate green innovation behavior, the manufacturer tends to align its behavior with government systems or social expectations and decide to pursue green innovation, with the aim to alleviate its development pressure. This decision helps to win government supports in technology, personnel, capital, and taxation and wins the recognition of shareholders.

It can also be seen from Figure 1 that, the manager (especially senior executive), after selecting strategic legitimate behavior of green innovation under the cognition of efficiency logic, will make one of two different green innovation decisions, namely, creation and control, in view of the scope of resource optimization and integration, as the enterprise takes the subjective initiative to perform its social and environmental responsibilities through green innovation. Specifically, creation is the green innovation decision of the manager that influences and partially reshapes the existing environmental systems, industry norms, and public perception by optimizing and integrating the resources controlled by the enterprise. In this case, the enterprise mainly builds a green recycling system for product manufacturing and relies on the system to serve consumers. The environmental operations will win the recognition from consumers and stakeholders and give the enterprise a sustainable competitive advantage. Control is the green innovation decision of the manager that seeks to fundamentally reform the existing environmental systems, industry norms, and public perception by rationalizing the allocation, optimization, and integration of internal and external resources of the enterprise, according to its embodied cognition. In this case, the manufacturer pursuing green innovation no longer eyes profit growth alone, but tries to replace the traditional non-ecological production model with a model that harmoniously coexists with the environment, while meeting the consumer demand for ecological stability. In addition, the enterprise will actively convey its own values, concepts, and progress of green innovation to the society. This would drive the green transformation of the whole industry and promote the low-carbon development of the whole industry chain. Under the cognition of efficiency logic and the selection of strategic legitimate green innovation behavior, the manufacturer tends to exert its subjective initiative and actively implement its social and environmental responsibilities, when manufacturer makes the decision on green innovation. During the pursuit of green innovation, the enterprise would organically integrate its own resources and externally acquired resources and occupy an advantageous position by its resource advantage. In this way, the enterprise could become a leader in green innovation and substantially enhance its core competitiveness.

## Conclusion and Suggestions

Through the research, it can be found that personal differences lead to varied embodied cognition of manager, which could affect the behavior selection of manufacturer green innovation. Driven by cognition-behavior selection of the manager, the manufacturer will make different green innovation decisions. Therefore, the following suggestions are proposed. The first is to integrate manager’s embodied cognition and green innovation behavior selection under its effect to make suitable green innovation decisions. The second is to gradually shift the cognition of manager (especially senior executives) from institutional logic to efficiency logic and then make the strategic legitimate behavior selection for green innovation exerting their subjective initiative. On this basis, making matching green innovation decisions so as to realize sustainable development through green innovation.

## Data Availability Statement

The original contributions presented in the study are included in the article/supplementary material, further inquiries can be directed to the corresponding author/s.

## Author Contributions

MH: contribution rate 57% including the overall idea of the manuscript, formulation of overarching research goals and aims, development of methodology, writing original draft, and writing review and editing. DZ: contribution rate 40% including writing original draft, writing editing, and visualization presentation of research results (drawing picture). DG: contribution rate 3% including material and sorting, references review, collection, sorting out, and manuscript format. All authors contributed to the article and approved the submitted version.

## Conflict of Interest

The authors declare that the research was conducted in the absence of any commercial or financial relationships that could be construed as a potential conflict of interest.

## Publisher’s Note

All claims expressed in this article are solely those of the authors and do not necessarily represent those of their affiliated organizations, or those of the publisher, the editors and the reviewers. Any product that may be evaluated in this article, or claim that may be made by its manufacturer, is not guaranteed or endorsed by the publisher.
